# Beyond Confinement: A Systematic Review on Factors Influencing Binge Drinking Among Adolescents and Young Adults During the Pandemic

**DOI:** 10.3390/jcm14051546

**Published:** 2025-02-25

**Authors:** Andrea Merino-Casquero, Elena Andrade-Gómez, Javier Fagundo-Rivera, Pablo Fernández-León

**Affiliations:** 1 Cruces University Hospital, Osakidetza-Basque Health Service, 48903 Bizkaia, Spain; 2 Department of Nursing, Faculty of Health Sciences, University of La Rioja, 26004 Logroño, Spain; 3 Red Cross University Nursing Centre, University of Seville, 41009 Seville, Spain; 4 School of Doctorate, University of Seville, 41009 Seville, Spain

**Keywords:** binge drinking, alcohol abuse, alcohol consumption, the COVID-19 pandemic, adolescents, young adults, risk factors, prevention, wellbeing, public health

## Abstract

**Objectives**: This study aimed to enhance the understanding of factors influencing changes in binge drinking (BD) behavior during the COVID-19 pandemic, with a particular focus on its impact on the health of individuals aged 12 to 25 years. **Methods**: A systematic review was conducted, encompassing studies published between January 2020 and September 2024. Articles were retrieved from PubMed, Web of Science, and Scopus, following PRISMA guidelines and the Joanna Briggs Institute (JBI) review protocols. Inclusion criteria targeted studies focusing on BD during the COVID-19 pandemic in adolescents or school-aged individuals without specific medical conditions. Exclusions included studies limited to a single gender, ethnicity, or profession, as well as doctoral theses and editorials. JBI tools were used to assess the quality of the selected studies. **Results**: From 33 studies (19 cross-sectional and 14 longitudinal), trends in BD during the pandemic varied: 2 studies reported an increase, while 21 indicated a decrease. Key factors linked to increased BD included pandemic stressors (e.g., isolation, social disconnection and non-compliance with restrictions), psychosocial issues (e.g., depression, anxiety, boredom, and low resilience), prior substance use, and sociodemographic variables (e.g., low education, economic extremes, living arrangements, and limited family support). Female gender and academic disengagement were also risk factors. Conversely, factors like stay-at-home orders, fear of contagion, family support, studying health sciences, and resilient coping strategies contributed to reduced BD. Other variables, such as pandemic stress and self-efficacy, had inconsistent effects. **Conclusions**: Factors contributing to increased BD included pandemic-related stress, mental health conditions, and unhealthy habits, while protective factors included stay-at-home orders, social support, and resilient coping. The study highlights the need for effective prevention and intervention strategies, emphasizing a holistic approach in healthcare, early detection, and tailored interventions, particularly for vulnerable groups such as adolescents.

## 1. Introduction

Alcohol consumption is a considerable public health concern that plays an important role in our society and culture. Based on the latest Global Status Report on Alcohol and Health and Treatment of Substance Use Disorders [1], 2.6 million deaths were attributable to alcohol consumption in 2019, representing 4.7% of all deaths in that year. The WHO European Region continues to have the highest level of per capita alcohol consumption in the world [2], despite a decrease from 12 L in 2000 to 9.5 L in 2019, which corresponds to a decrease of 10% in 2010 and 21% in 2019. It should also be noted that Europe is the WHO region with the highest percentage of deaths attributable to alcohol consumption (10.1%) [3]. In Spain, an estimated 13,887 deaths were attributable to alcohol consumption in 2021 [4]. Moreover, over 90% of the population aged 15 to 64 report having consumed alcohol at some point in their lives [5]. These figures highlight alcohol consumption as a significant public health concern, particularly considering that they account only for the direct effects on consumers and do not include harm to third parties [6].

Adolescents and young adults are a particularly vulnerable population about developing harmful patterns of alcohol consumption, such as binge drinking (BD), which may emerge during this developmental stage and become established. Binge drinking is defined as the consumption of a large quantity of alcohol within a single occasion—typically five or more alcoholic drinks for men and four or more for women—resulting in a state of intoxication, which is subsequently followed by periods of abstinence [7]. This drinking pattern has severe long-term consequences, including traffic accidents, violence, homicide, suicide, early and high-risk sexual behavior, academic and occupational failure, mental disorders, and delinquency. Additionally, it leads to short-term effects such as alcohol poisoning, loss of consciousness, and memory blackouts [8]. Globally, the prevalence of BD was 18.2% in 2018, which amounts to one billion people worldwide [3]. Furthermore, it shows high picks of frequency and more percentages of episodes in adolescents and young adults [9,10,11,12].

Since December 2019, the emergence of the severe acute respiratory syndrome coronavirus 2 (SARS-CoV-2), the primary causative agent of the coronavirus disease 2019 (COVID-19) pandemic, has led to the declaration of a public health emergency of international concern. This pandemic has resulted in more than 776 million confirmed cases and over 7 million deaths [13]. The global nature of this pandemic has profound economic, social, and public health implications [14]. In this context, health behaviors play a crucial role in sustaining health and preventing both infectious and non-infectious diseases. Although individuals should have pursued multiple protective behaviors and avoid risky behaviors, adherence to behavioral recommendations during COVID-19 was variable [15]. Specifically, alcohol consumption and BD behavior during the COVID-19 pandemic had a significant influence with different and even opposite results between diverse countries and population groups [13]. Several studies conducted during the pandemic have reported divergent findings, indicating that BD patterns among adolescents and young adults were influenced by lockdown measures in two distinct ways. While some individuals exhibited an increase in these behaviors [16,17], others reported a decline [18,19].

Although the existing evidence suggests various factors that may contribute to the increase or decrease of BD among teenagers and early adulthood population during the COVID-19 pandemic, there appears to be a lack of systematic reviews that compile and standardize this information. Consequently, this study aims to bridge this gap by summarizing and updating which factors modulate the increase or reduction of the alcohol BD pattern during the COVID-19 pandemic. It provides a comprehensive overview of the relevant literature and examines its implications for the health of adolescents and early adults.

## 2. Materials and Methods

### 2.1. Protocol and Registration

The review process was based on the Joanna Briggs Institute (JBI) guidelines for systematic reviews [20] and followed the PRISMA statement [21] (Table S1. PRISMA checklist). Furthermore, this systematic review was registered in PROSPERO (code: CRD42024552338).

### 2.2. Design

For this systematic review, the PICO approach was adopted to structure the review questions and define eligibility criteria:

P (population): adolescents and young adults;
I (intervention): binge drinking during the COVID-19 pandemic;
C (comparison): binge drinking before the COVID-19 pandemic;
O (outcome): modulating factors of the increase/reduction of the BD.

In adolescents and young adults (P), what were the modulating factors of the increase/decrease in BD (O) before (C) and during the COVID-19 pandemic (I)?

### 2.3. Information Sources and Search Strategy

The searches were carried out in September 2024 in the Pubmed, Web of Science, and Scopus (Science Direct) databases by two reviewers. A third reviewer acted in case of discrepancies. Articles conducted on humans and published between January 2020 and September 2024 were selected. The search strategy included MeSH terms: (adolescent* OR youth* OR teen* OR student*) AND (“binge drinking” OR “binge alcohol consumption”) AND (COVID-19 OR SARS-CoV-2 OR “COVID-19 pandemic” OR “Coronavirus Disease 2019”).

The search strategy in each database is presented in Table 1.

### 2.4. Eligibility Criteria

The research inclusion criteria were as follows: (1) original studies (quantitative or qualitative design) that addressed binge drinking during the COVID-19 pandemic; and (2) studies carried out in adolescents and scholar-aged individuals, without specific pathologies or medical conditions. The exclusion criteria for the research were as follows: (1) documents focused on populations exclusively of one gender, profession, or ethnicity; (2) systematic reviews, meta-analyses, doctoral dissertations, brief reports, conference proceedings, commentaries, and editorial articles; and (3) studies with low quality in methodological assessment according to the Joanna Briggs Institute (JBI) critical appraisal tools.

### 2.5. Selection and Data Collection Process

**Figure 1:**
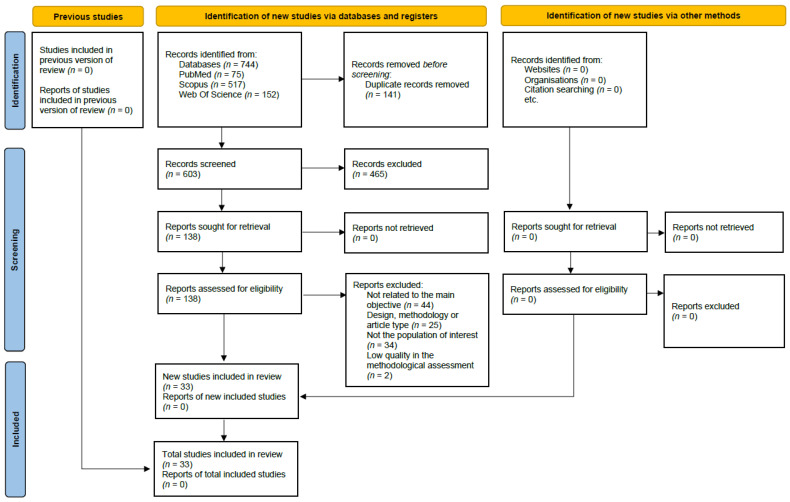
PRISMA flowchart.

A total of 744 publications were identified through database searches. From this initial pool, 141 duplicate entries were removed. Following a review of titles and abstracts, 465 articles were eliminated for failing to align with the specified objectives and research questions. Subsequently, 138 reports were evaluated for eligibility, resulting in the exclusion of 105 reports for various reasons (not related to the main objective (*n* = 44); design, methodology, or article type (*n* = 25); not the population of interest (*n* = 34); low quality in the methodological assessment (*n* = 2). Finally, 33 studies were included in the review. The selection process and the rationale for exclusions are illustrated in the PRISMA flowchart presented in Figure 1.

### 2.6. Study Risk of Bias Assessment

The Joanna Briggs Institute (JBI) critical appraisal tools have been designed for its use in systematic reviews, enabling the assessment of the reliability, relevance, and outcomes of published works. These critical appraisal tools were developed by the JBI and its collaborators and approved by the JBI Scientific Committee following extensive peer review. The quality of all selected articles was examined using different checklists, depending on the type of study. In this regard, the tool for analytical cross-sectional studies and the tool for cohort studies were used [22]. Studies scoring below half of the maximum possible value (i.e., 4/8 or lower for analytical cross-sectional studies and 5/11 or lower for cohort studies) were categorized as low quality and were thus removed from the final selection (Tables S2 and S3).

### 2.7. Synthesis Methods

In this systematic review, a narrative synthesis approach was employed to qualitatively integrate and interpret the findings from the selected studies. This method facilitated a comprehensive and coherent analysis by identifying key themes, patterns, and inconsistencies within the literature. By synthesizing diverse sources without the application of statistical meta-analysis, this approach enabled the contextualization of evidence, the identification of knowledge gaps, and the provision of meaningful insights into the research topic.

## 3. Results

### 3.1. Study Selection and Characteristics

A total of 33 studies were chosen for the systematic review after screening based on the inclusion and exclusion criteria, title, abstract, and full text.

Table 2 presents specific information about the 33 included studies [16,17,18,19,23,24,25,26,27,28,29,30,31,32,33,34,35,36,37,38,39,40,41,42,43,44,45,46,47,48,49,50,51].

The selected research studies were conducted across various countries, including the United States [18,19,23,31,33,34,37,38,44], Italy [25], Germany [26], Australia [27], Canada [16,28,29,30,40,45], France [32,36,46], Guatemala [35], United Kingdom [17], Spain [39], Netherlands [41,49], Turkey [42], Israel [43], Belgium [47], Denmark [48], Portugal [50], and Switzerland [51], and included one nationwide study [24].

The studies focused on specific periods of the pandemic. For instance, some studies examined a single month, such as April 2020 [24] and May 2020 [26]. Other investigations covered a range of months within 2020, including March to May [25,46], April to May [19,32,47], April to June [49], February to March and July to August [34], April to July [41], May to June [42], May to July 2020 [40], April to September [43], April to November [37,50], August to November [48], and October to December [33]. Additionally, some studies spanned broader timeframes, such as 2009 to 2021 [31], 2015 to 2020 [17], 2017 to 2020 (including May–June 2020) [27], 2017 to 2021 [29], 2018 to 2021 [28,30], 2019 to 2020 [18,35,39], and 2020 to 2021 [16,23,36,38,44,45,51].

The studies indicated that the estimated mean age was 21 years, with an age range varied from 12 to 25 years. Furthermore, the average proportion of female participants across all studies was found to be 65%.

The studies indicate a varied prevalence of BD, with some reporting an increase in trends [16,17], while others have noted a decline [18,19,24,25,27,28,31,32,35,36,37,38,39,41,42,43,46,47,48,49,50].

The research includes 19 cross-sectional studies [19,23,24,25,26,31,32,33,34,35,36,40,41,42,43,46,47,48,49] and 14 longitudinal studies [16,17,18,27,28,29,30,37,38,39,44,45,50,51].

### 3.2. Risk of Bias in Studies

The included studies met the minimum threshold according to the JBI critical appraisal tools, achieving a rating of moderate to high quality. For cross-sectional studies, the obtained scores were 5/8 [33,34,35], 6/8 [19,23,24,31,36,42,48,49], 7/8 [25,26,32,40,43,46,47], and 8/8 [41]. For cohort or longitudinal studies, the scores were 7/11 [38,45], 8/11 [18,27,37,44], 9/11 [16,17,39,51], and 10/11 [28,29,30,50].

### 3.3. Results of Individual Studies

Longitudinal research provided more significant insights into the evolution of BD over time and the causal relationships between various factors and behaviors. These studies revealed fluctuations in BD patterns throughout different phases of the pandemic, highlighting consistent trends and identifying protective or risk factors. Notable findings included an initial decrease in BD, primarily associated with reduced social opportunities due to lockdowns and restrictions, followed by a resurgence in the second year of the pandemic, particularly among men [29] and older adolescents, linked to feelings of boredom [27,37], loneliness, and depression [28]. The longitudinal studies identified several consistent predictors of BD, including psychosocial factors such as depression [28,30] and anxiety [17,28,30,44,45,51]; a lack of coping strategies and low academic engagement [18]; prior excessive alcohol consumption as a significant risk factor [45]; and insufficient social and familial support [16,37,45,50,51], which contributed to the increase in BD. These findings emphasize the intricate interplay between social conditions [44,50].

Considering both longitudinal and cross-sectional studies, several factors associated with the COVID-19 pandemic have been identified as contributing to an increase in BD. These include the direct effects of COVID-19 infection [33], experiences of isolation [19,23], disconnection from social environments [19,23,34], loneliness [23,42], stress related to COVID-19 [37,43,47], social restrictions resulting from the pandemic [40], and non-compliance with these restrictions [40,49]. Several factors contributing to the increase in BD associated with psychosocial health include: lack of coping strategies [18,19], depressive symptoms [26,30,32,41,42,46,47], anxiety symptoms [30,32,44,50,51], boredom [26,27,37], risk of suicide [32], low resilience [41], and having a previous mental health diagnosis [45].

Various factors can be identified that connect BD to other health-related behaviors. These include a lifetime of regular smoking [23,32,43,44], binge drinking prior to the pandemic [18,41,50,51], lack of physical activity [32], using substances (i.e., cannabis) [18,43], and other risk behaviors [43].

The sociodemographic variables contributing to various outcomes include educational attainment such as primary or middle school education [25,44], vocational training [39], and bachelor studies [49]. Additionally, economic status plays a significant role, encompassing both low [25,45,49] and high economic status [39,43]. Social living arrangements, whether living alone, with a partner, or with friends, are also relevant factors [25,35,37,41,45,46,49,50,51]. Furthermore, involvement in Greek life and the presence of friends or social groups [19,35,36,41,51], and being in a complicated relationship [26,49] are determinants. Other notable influences include low family support [43], limited recreational activities [27], and the impact of distance learning and academic disengagement [18,42,47].

Numerous studies have indicated a correlation between female gender and an increased risk of BD during the COVID-19 pandemic [17,28,31]. Conversely, other research has identified male gender as a contributing factor to the risk [29,41,43,46,49,51]. Additionally, both younger [29,51] and older adolescents [39,41,43,44,49] have been associated with heightened levels of BD. Furthermore, the second year of the pandemic was related to an increase in BD compared to the year 2020 [29].

Several factors associated with COVID-19 have been linked to a reduction in BD. These include confinement measures and the implementation of initial stay-at-home orders [16,24,48,49,50]. Additionally, social distancing practices played a role in this decrease [18,46,47,50]. The presence of supportive relationships during this period [19,42] also contributed positively. Furthermore, the anxiety surrounding potential infection and the risk of transmitting the virus to others was a significant factor [46]. Resilient coping strategies were found to correlate with a decline in BD [18]. Moreover, individuals who did not have a history of alcohol consumption were also observed to have a lower risk of experiencing BD [28].

A reduction in BD has been associated with several sociodemographic factors, including residing with family members [25,46] and being enrolled as a student [28,46], with a particular emphasis on those studying health sciences [46]. Additionally, individuals from lower economic backgrounds have also been linked to a decrease in BD [47].

Conversely, the factors of being female [26,27,42] and younger [26,27,28] appeared to mitigate the incidence of BD, whereas [45] reported a decreased risk of BD among older individuals.

Additionally, various studies have identified certain variables that did not correlate with fluctuations in BD, including pandemic-related stress and social support [25,48], smoking and cannabis use [26], pandemic-related stressors [18,28,43], low economy [38], physical activity [43], support from friends [43], as well as low resilience and low self-efficacy [51].

## 4. Discussion

This systematic review highlights a range of risk factors that contribute to the prevalence of the BD behavior. Evidence suggests that BD may be linked to various underlying causes, including the impact of COVID-19 restrictions, mental and behavioral health disorders, familial difficulties, and socioeconomic challenges.

Numerous factors linked to the pandemic have emerged as significant influences, including COVID-19 infection [33], experiences of isolation [2,19], social disconnection [19,23,34], feelings loneliness [3,42], stress stemming from the health crisis, social restrictions [37,43,47], and instances of non-compliance with pandemic restrictions [40,49]. Furthermore, the second year of the pandemic saw a rise in BD compared to 2020 [29].

Several factors contribute to a low psychosocial health, including insufficient coping mechanisms [18,19], depressive symptoms [26,30,32,41,42,46,47], anxiety [30,32,44,50,51], feelings of boredom [26,27,37], suicide risk [32], low resilience [41], and pre-existing mental health conditions [45]. Research suggests that neuroticism, boredom proneness, and type D personality—characterized by a heightened sensitivity to negative emotions and a tendency to suppress emotional expression in social contexts—may serve as inefficient strategies for dealing with emotional problems [52]. Additionally, another study identifies BD as a potential strategy to mitigate depressive or anxious moods which can create a bidirectional cycle where excessive alcohol consumption and emotional distress reinforce each other [53]. In relation to mental health, social behavior disorders or ADHD issues may also correlate with BD [54]. Sensation-seeking behavior, along with low self-control and impulsivity—traits more commonly observed in adolescents and males—can lead to difficulties in resisting urges, such as the consumption of alcohol [52]. Moreover, BD is significantly associated with an increased risk of suicide and suicide attempts [54] and has been connected to the onset of anxiety and depression [55].

Various additional habits, including habitual smoking [23,32,43,44], high levels of alcohol intake before the onset of the pandemic [18,41,50,51], lack of physical activity [32], substance use (such as cannabis), and other risky behaviors [18,43], were also identified as contributing factors for BD. Research has indicated a correlation between self-identifying as a social smoker and instances of BD [56]. Similarly, cannabis consumption appears to exacerbate this association, particularly among college students and undergraduates. Furthermore, binge drinkers who also use cannabis exhibit diminished neuropsychological performance, notably characterized by deficits in episodic memory [57,58,59].

Sociodemographic factors contributing to the phenomenon include educational attainment (such as middle school, bachelor’s degrees, and vocational training) [25,39,44,49], low socioeconomic status [25,45,49], and, conversely, high socioeconomic status [39,43]. Additional factors encompass living alone [25,35,37,41,45,46,49,50,51], limited familial support [43], a lack of engaging activities [27], the prevalence of distance learning, and academic disengagement [18,42,47]. The correlation with extraversion, potentially linked to peer influence, underscores the considerable impact of the social context on BD [52,60]. Consequently, it is significant to note how both feelings of loneliness and social isolation have variably affected BD during the pandemic. Specifically, these factors have led to an increased prevalence of BD among individuals exhibiting depressive or anxious tendencies, as well as those experiencing heightened loneliness. Conversely, a general trend observed across studies indicates a substantial reduction in BD, attributed to diminished social interactions and fewer opportunities for alcohol consumption. Other relevant variables include family dysfunction [60], parental drinking behaviors [53], and living independently from one’s family [61], all of which were associated with BD prior to the onset of the COVID-19 pandemic. Furthermore, relatively high income [62,63] or the availability of disposable income, which facilitates access to alcohol, particularly among university students during weekends, has also been linked to BD [60].

Our research outlines various factors associated with a decrease of BD in the context of COVID-19. Pandemic-related factors include confinement and stay-at-home orders [16,24,48,49,50], social distancing [18,46,47,50], fear of contagion [46], and resilient coping strategies [18]. Feeling supported by loved ones [19,42] and not having been a prior alcohol consumer [28] are also linked to lower levels of BD.

In terms of sociodemographic factors, living with family [25,46], being a student (particularly in health sciences) [28,46], or having a low economic status are associated with reduced BD levels [47]. Furthermore, it has been observed that the risk of BD tends to decline among older adolescents [45]. Factors such as religiosity [60,63], social support from family and friends [63], and parental control [53,60,61] have been identified as protective factors for BD before the pandemic, and thus, these factors remained active from 2020 onwards.

Numerous studies have examined the influence of sociodemographic factors, particularly sex, on the prevalence of BD during the COVID-19 pandemic. Some research indicates that being female is associated with a heightened risk of BD during this period [17,28,31]. Conversely, other studies suggest that being male may correlate with an increased risk [29,41,43,46,49,51]. This finding aligns with earlier research that identified a higher prevalence of BD among males compared to females [64].

The variable of age similarly demonstrates a correlation with BD, as both younger [29,51] and older adolescents [39,41,43,44,49] have been associated with this behavior. Individuals within the 18–29 age range exhibited a higher likelihood of reporting fluctuations in BD—both increases and decreases—during the COVID-19 pandemic, suggesting that distinct mechanisms may underlie these divergent trends. Research indicates that the psychological ramifications of the pandemic may have disproportionately affected young and middle-aged adults in comparison to older individuals [65,66,67,68]. Furthermore, studies reveal that adolescents who initiate regular alcohol consumption prior to the age of 15 are up to four times more susceptible to developing alcohol dependence than those who start drinking at a later age [54]. This finding supports the notion that alcohol consumption tends to increase in both frequency and quantity as individuals age [69,70].

Longitudinal studies conducted during the COVID-19 pandemic have provided valuable insights into the evolving patterns of BD. A comprehensive analysis indicates that, while there was a notable decline in BD prevalence during the initial lockdowns, particularly in the first year, a resurgence was observed in the second year of the pandemic [16]. The initial reduction in BD was primarily linked to diminished social opportunities [18], which were a consequence of restrictions such as lockdowns and stay-at-home orders [17]. Factors including cohabitation with family and a decrease in social interactions were identified as protective elements [18]. However, as restrictions began to relax in the later stages of the pandemic, an increase in BD patterns was particularly evident among males and older adolescents [30,51]. Research has indicated that feelings of boredom [37], loneliness [50], and depression [30] played significant roles in influencing BD behaviors, with some studies noting that women experienced higher rates of BD initiation and escalation compared to men during the pandemic [30]. Furthermore, younger individuals and students who had pre-existing BD habits were more inclined to resume or amplify their BD as restrictions were lifted [27]. Conversely, when examining the interplay of sex and age, being female [27,28] appeared to mitigate the risk of BD, although [45] noted a reduced risk of BD among older adolescents during the second year of the pandemic compared to 2020. The variability observed across different demographic groups and time periods highlights the intricate relationship between pandemic-related stressors, mental health, and alcohol consumption behaviors. These findings underscore the necessity for targeted interventions that can effectively address the fluctuating nature of BD during extended crises.

### 4.1. Future Approach and Implications

Considering the results, particularly those from longitudinal studies, future measures aimed at curbing excessive alcohol consumption should take several key factors into account, especially in the context of the COVID-19 pandemic and its aftermath.

First, psychosocial support is essential [71]. Providing mental health support, especially during periods of isolation or social restrictions, could mitigate the increase in alcohol consumption associated with depression, anxiety, and boredom. The prolonged uncertainty and emotional distress caused by the pandemic have intensified these psychological challenges, making it even more crucial to ensure accessible mental health resources [72]. Additionally, promoting resilient coping strategies may help individuals manage pandemic-related stress without resorting to BD, particularly as people continue to adapt to post-pandemic societal changes [18,28].

Social support, including peer support, has proven to be an effective resource for improving mental health during the pandemic. Family support also plays a crucial role, as living with family has been identified as a protective factor against BD [63]. During lockdowns, the home environment became a central influence on behavior, either reinforcing protective factors or, in some cases, exacerbating stressors. Encouraging participation in recreational activities could further reduce BD associated with boredom, which was a major contributor to increased alcohol use during periods of strict confinement and limited social interaction [73].

Moreover, sex- and age-specific interventions should be developed, given that these demographic factors influence BD patterns. The pandemic highlighted distinct vulnerabilities among different groups, with studies showing that men and older adolescents were particularly prone to increased alcohol use during lockdowns [29]. Tailoring interventions to address these specific needs is essential. Additionally, the social environment must be considered, as peer influence is a significant factor [16,37,45,50,51]. The shift from in-person to virtual socialization during the pandemic altered drinking behaviors, and interventions should address both online and offline peer pressures [74].

Another critical aspect is distance education, as challenges related to remote learning and academic disengagement have been linked to an increase in BD [18,42,47]. The transition to online education during the pandemic disrupted students’ routines, reduced their access to structured environments and contributed to feelings of disconnection, all of which may have powered unhealthy coping mechanisms such as excessive alcohol consumption. Addressing these educational challenges and fostering engagement in academic activities can serve as a protective factor [75].

Additionally, early intervention is necessary to prevent alcohol consumption at a young age, as early alcohol use has been associated with a higher risk of developing addiction-related problems. The pandemic underscored the importance of early preventive measures, given that many adolescents experienced increased exposure to alcohol due to changes in parental supervision and home dynamics during lockdowns. Initiating alcohol consumption at an early age has been found to be associated with an increased risk of developing addiction problems. This highlights the importance of directing intervention and approach efforts towards adolescents; these measures would be implemented in both primary care and pediatric services, as well as educational institutions [76,77,78,79] and those professionals specialized in the prevention of addictive behaviors [80].

A notable association has been identified between BD and attitudes associated with drunkorexia, as well as the use of cocaine and novel psychoactive substances [81,82,83]. Research indicates that fasting BD and intoxication serve as predictors of drunkorexic behaviors [84]. Furthermore, the reduction in physical activity during the pandemic may have exacerbated these behaviors in two significant ways. Individuals might have attempted to offset the perceived decrease in caloric expenditure by resorting to disordered eating practices, such as fasting or omitting meals prior to alcohol consumption, which are characteristic of drunkorexia [82]. Additionally, BD, which is closely associated with drunkorexia, may have intensified due to increased emotional stress and boredom experienced in times of social distancing, as documented in various studies examining alcohol consumption during the pandemic [26,27,37,43,47,83,85].

Furthermore, the pandemic not only influenced drinking behaviors in the short term but may also have long-term effects on alcohol consumption patterns. Thus, it is crucial to recognize that prevention and intervention strategies should adopt a holistic approach, considering the physical, mental, and social consequences of BD, including the increased likelihood of alcohol dependence. Despite often being misconceived as a lifestyle choice tied to university culture, various studies have explored the interplay between food, alcohol, and physical activity, particularly in young adults [86,87,88]. Nonetheless, results are not conclusive, and it is necessary to continue with further research to understand whether binge behaviors (eating or drinking) interaction [32]. This way, future research should invest in time and resources for health promotion from an early age to mitigate the pattern of BD consumption among young people [52]. It has been identified the need to expand the preventive approaches beyond the individual, leading to healthier habits and a better quality of life in the short and long term. Implementing environmental measures in places frequented by young people, such as bars, pubs, and mass events, as well as interventions in the family, educational, and media environments [89], are effective strategies to guarantee the success of these approaches. Addressing these risks requires a comprehensive approach that integrates public health policies, mental health initiatives, and community-based support systems to mitigate the lasting impact of the pandemic on alcohol use behaviors.

### 4.2. Limitations

This study acknowledges variability in the definition of BD across included studies. This inconsistency may hinder the comparability and synthesis of results, although the operational definitions of BD were explored in all the included studies to assure the consistency of the results. In addition, data collection bias is addressed in this study, as self-reported data from included studies may underestimate actual alcohol consumption due to social desirability bias or memory inaccuracies. Sampling bias is also acknowledged as some included studies suffered from small sample sizes, reducing statistical power and generalizability. Finally, factors such as pandemic-related stress, social support, and self-efficacy were reported inconsistently across studies, complicating a cohesive understanding of their role in BD. The cross-sectional design of most studies may have contributed to this limitation. Not all results obtained in the study hold the same level of significance, and more relevant conclusions can be drawn from certain factors, depending on the methodology employed. While cross-sectional studies have provided valuable insights, their ability to establish causal relationships is limited. For instance, although some cross-sectional studies reported a higher risk of BD among females during the pandemic, others suggested the opposite. These discrepancies may arise from variations in study populations or the inherent limitations of cross-sectional studies in capturing shifting behavioral patterns during the pandemic.

## 5. Conclusions

This systematic review included 33 studies, with 19 cross-sectional and 14 longitudinal studies. Findings on BD during the COVID-19 pandemic are clearly unbalanced, as two studies reported an increase in BD prevalence, while 21 reported a decrease. Factors contributing to increased BD included pandemic-related stressors like COVID-19 infection, isolation, loneliness, and non-compliance with social restrictions. Psychosocial factors such as depression, anxiety, lack of coping strategies, and pre-existing mental health conditions were significant, as were habits like prior excessive alcohol use, smoking, cannabis consumption, and physical inactivity. Sociodemographic factors like low education, varying economic status, and limited family support were also linked to BD.

Conversely, some pandemic-related factors were also associated with reduced BD, including stay-at-home orders, fear of contagion, resilient coping, and feeling supported by loved ones. Protective factors included living with family, studying health sciences, and having a low economic status. Variables like pandemic-related stress and self-efficacy showed inconsistent relationships with BD. These findings underscore the importance of understanding the ineffective coping strategies that influence BD behaviors during crises such as the pandemic.

The study concludes that effective prevention and intervention strategies are essential, focusing on the physical, mental, and social consequences of BD, including the increased likelihood of alcohol dependence. A holistic approach to BD in healthcare settings is needed, integrating early detection, risk factor evaluation, and tailored interventions, particularly for vulnerable groups like adolescents. Training healthcare professionals is crucial for effectively addressing BD behaviors and implementing preventive measures.

## Figures and Tables

**Table 1:** Search strategies in databases.

Database	Search Strategy	Search Date	Outcomes
PubMed	(adolescent* OR youth* OR teen* OR student*) AND (“binge drinking” OR “binge alcohol consumption”) AND (COVID-19 OR SARS-CoV-2 OR “COVID-19 pandemic” OR “Coronavirus Disease 2019”).	2020–2024	75
WOS	(adolescent* OR youth* OR teen* OR student*) AND (“binge drinking” OR “binge alcohol consumption”) AND (COVID-19 OR SARS-CoV-2 OR “COVID-19 pandemic” OR “Coronavirus Disease 2019”).	2020–2024	152
Scopus	(adolescent OR youth OR teen OR student) AND (“binge drinking” OR “binge alcohol consumption”) AND (COVID-19 OR “COVID-19 pandemic”).	2020–2024	517
Total			744

**Table 2:** Study selection.

Reference and Context	Data Collection	Objective of the Study	Type of Study	Participants	Methods	Main Findings	Quality of Studies
Ahuja et al., 2024. USA. [23]	2020–2021	To examine the relationship between loneliness and varying levels of alcohol consumption among college students in a rural region of the United States during the COVID-19 pandemic.	Cross-sectional study	Sample of (*n* = 310) people of a same region; age range: 18–25 (mean = 21.3; SD = 1.9 years); 69.7% women; 30.3% men.	UCLA-3 loneliness scale; binge drinking: “During the last 12 months, how often did you have 5 or more (for males) or 4 or more (for females) drinks containing any kind of alcohol in within a two-hour period?” logistic regression analysis to assess the association between loneliness binge drinking, heavy alcohol use, and weekly alcohol use.	The prevalence of binge drinking was found to be 11.0% (*n* = 34) of the sample. The COVID-19 pandemic has resulted in increased feelings of isolation and disconnection among many students, rendering them more vulnerable to experiencing loneliness. Severe loneliness was significantly associated with a higher likelihood of binge drinking (AOR = 2.96; 95% CI: [1.16, 7.51]). Furthermore, lifetime regular smoking was also correlated with binge drinking (AOR = 4.68; 95% CI: [1.85, 11.81]).	6/8
Ammar et al., 2020. International [24]	April 2020	To elucidate the behavioral and lifestyle consequences (on physical activity and nutrition behaviours) of COVID-19 restrictions.	Cross-sectional study	Sample of (*n* = 1047) people from Asia, Africa, Europe, America and others; age range: 18 to +55 (55.1% from 18 to 35 years; 35.1% from 36 to 55 years); 54% women and 46% men.	International online survey on mental health and multi-dimensional lifestyle behaviors before and during home confinement; SDBQ-L (Question 4. Alcohol Binge Drinking); *t*-tests and effect size (Cohen’s d) were used.	Prevalence of binge drinking included 12.31% (*n* = 129) of the sample before COVID-19 and 6.79% (*n* = 71) during confinement. The COVID-19 home confinement had a positive healthy effect on alcohol binge drinking, whose prevalence decreased significantly (t = −12.16, *p* < 0.001; effect size: d = 0.58). The responses that indicated alcohol binge drinking were lower during home confinement (5.4% for sometimes, 1.2% for most of the time, and 0.2% for always). Alternatively, COVID-19 home confinement had a negative effect on all levels of physical activity, increased sedentarism and revealed unhealthy patterns of food consumption.	6/8
Bianchi et al., 2021 Italy [25]	March–May 2020	To investigate binge drinking and binge eating behaviors among emerging adults in Italy during the COVID-19 lockdown, while examining potential changes in these behaviors in comparison to the pre-pandemic period.	Cross-sectional study	Sample of *n* = 1925 emerging adults; mean age: 24.18 (SD = 2.75 years); age range: 18–29 years; 71.9% women and 28.1% men.	BD: “During the last 30 days before the introduction of national lockdown, how many times have you drunk 5 or more alcoholic drinks—4 if you are female—on one single occasion?”; and during the current quarantine (i.e., “During the current period of lockdown, how many times have you drunk 5 or more alcoholic drinks—4 if you are female—on one single occasion?”). Three MANOVA analyses were conducted.	During the quarantine period, the incidence of binge drinking (BD) episodes demonstrated a significant decline. Nonetheless, 320 participants (16.6%) reported experiencing BD, while 84 participants (4.4%) indicated an increase in the frequency of BD during the lockdown. Participants with primary or middle school education, as opposed to those with higher educational attainment, along with individuals from lower economic backgrounds, were more likely to belong to the BD increase group. Conversely, participants residing with their families exhibited a reduced likelihood of reporting BD; those living alone, with partners, or with other individuals during the lockdown were more frequently categorized in the BD increase group. No statistically significant differences were observed concerning pandemic-related stress or social support among the BD increase groups.	7/8
Bonar et al., 2021 USA [19]	April–May 2020	To examine first-year college students’ binge drinking habits after their university’s pandemic-related suspension of in-person operations and investigate differences in demographic, psychosocial and COVID-19-related variables among students.	Cross-sectional study	Students from a single university campus (*n* = 741); mean age = 18.05 (SD = 0.22 years); 472 women (63.6%) and 269 men (36.3%).	COVID-19-related variables; sex-specific binge drinking frequency pre- and post-campus closure; 20-item Drinking Motives Questionnaire. Non-parametric paired *t*-test, and variable contrast using chi-square and F-tests were used.	The study examined binge drinking prevalence before and after the COVID-19 campus closure. Results showed a decrease in binge drinking frequency, with M pre = 1.54 (SD = 1.38) and M post = 0.72 (SD = 1.10). While 49.66% of students did not binge drink at either time, 6.75% maintained consistent binge drinking behavior. Notably, 39.41% reported reduced binge drinking post-closure, while 4.18% increased their binge drinking. Additionally, 19.35% shifted from abstaining from alcohol to binge drinking after the closure, and 33.22% who previously binge drank stopped entirely. Lower post-closure binge drinking was linked to coping motives and feelings of isolation, while higher levels were associated with Greek life involvement. Non-binge drinkers experienced significantly less isolation and loneliness due to COVID-19.	6/8
Busse et al., 2021 Germany [26]	May 2020	To investigate the impact of the COVID-19 pandemic, with particular emphasis on the lockdown period, on the engagement in health-related behaviors (HRBs) among university students in Germany	Cross-sectional study	Sample consisted on (*n* = 5021) students of four German universities; age range: 17 to 25; mean age = 24.4 (SD = 5.1 years); 69% women and 29% men.	A web-based survey using the HRB scale (with pre-during pandemic questions): (item 3) binge drinking, consisting on the question “How often do you have six or more drinks on a single occasion?”; 8-item Center for Epidemiologic Studies—Depression Scale. Descriptive analysis and multinomial logistic regression analyses were used. A latent transition analysis among substance use behavior (smoking, binge drinking, and cannabis use) was conducted.	A total of 45.8% of students (*n* = 4965) reported engaging in binge drinking both before and during the COVID-19 pandemic. Prior to the pandemic, 2% of students indicated binge drinking more than once per week, which increased to 4% during the pandemic. Nearly half of the participants acknowledged binge drinking before the pandemic, while 24.4% reported a decrease in such behavior during this period, and 5.4% noted an increase; however, 70.2% experienced no changes in their binge drinking patterns. Factors contributing to increased binge drinking included depressive symptoms, boredom, and complex relationships. Interestingly, women and younger individuals were more likely to reduce their binge drinking during the pandemic. Additionally, a significant co-occurrence of smoking, binge drinking, and cannabis use was observed among the student population.	7/8
Clare et al., 2021 Australia [27]	Four phases: 1. September 2017–July 2018; 2. September 2018–May 2019; 3. August 2019–January 2020; 4. May–June 2020.	To estimate the changes in alcohol consumption among young people during the COVID-19 restrictions and to analyze whether these changes varied by gender and pre-pandemic consumption levels	Prospective longitudinal cohort study	Subsample of a cohort (*n* = 443) of secondary school students; mean age: 19.7 (SD = 0.5 years); 60.8% women and 39.2% men; data from the Australian Parental Supply of Alcohol Longitudinal Study.	Sociodemographic: time, gender, and level of consumption prior to the pandemic. In addition, frequency and typical quantity of alcohol consumption, binge drinking, peak consumption, alcohol-related harm and drinking contexts. Frequency of binge drinking: participants who reported any alcohol consumption were asked how often they consumed ≥ 5 standard drinks.	During the COVID-19 restrictions, young Australians experienced a significant reduction in overall alcohol consumption, binge drinking, and alcohol-related harms. Binge drinking rates were stable prior to the pandemic but declined by 28% from February to May–June 2020. During this period, the proportion of participants abstaining from binge drinking increased from 35.3% to 50.0%, while those reporting sporadic binge drinking decreased from 50.6% to 40.8%. Additionally, the prevalence of weekly binge drinking fell from 14.1% to 9.2%. These changes in drinking patterns were observed across genders, though the decline in overall consumption was notably significant among women. The main motivations for binge drinking included feelings of boredom and limited engagement in activities.	8/11
Dumas et al., 2022 Canada [16]	Four phases: T1: April 2020; T2: August to September 2020; T3: January to February 2021; T4: June 2021.	To investigate adolescent substance use patterns throughout the pandemic, emphasizing the impact of stay-at-home orders and re-opening phases	Longitudinal cohort study	Sample of *n* = 1068 adolescent students; age range: 14–18 years; mean age = 16.95 (SD = 0.84 years); 76.7% women and 23.3% men.	The study included demographic questions and inquiries related to substance use during the pandemic. Binge drinking is operationally defined as the intake of four or more standard drinks for females and five or more for males in one sitting, where a standard drink is equivalent to 341 mL of beer, 142 mL of wine, 43 mL of liquor, or 341 mL of a premixed beverage.	Before the pandemic, the prevalence of binge drinking was 19.4%. The study identified variations in binge drinking rates across different lockdown phases: a low of 9.4% during the first lockdown (T1), a peak of 20.8% after restrictions were lifted (T2), a decrease to 17.8% during the second lockdown (T3), and a stabilization at 18.6% following the third lockdown (T4). Statistical analyses revealed that participants exhibited a significantly higher likelihood of abstaining from binge drinking during T1 compared to later phases, with increased abstinence from T2 to T4 (*p* < 0.001). Additionally, adolescents demonstrated a decreased propensity for binge drinking after the initial stay-at-home orders were lifted.	9/11
Fruehwirth et al., 2021 USA [18]	Wave I: October 2019 and February 2020 (prior to the pandemic). Wave II: June/July 2020.	To study how COVID-19 stress-related factors and changes in social engagement during the pandemic contributed to changes in alcohol use among first-year college students.	Longitudinal cohort study	Sample of *n* = 439 first-year, university students; age range: 18–20 years; mean age = 18.9 (SD = 0.018); 72% women, 28% men.	Youth Risk Behavior Surveillance System: analysis of prevalence and days of binge drinking. Associations between pre- and intra-pandemic stressors and social engagement were established. All models were estimated as a function of COVID-19 stressors/stress and social engagement variables.	Over the past 30 days, the prevalence of binge drinking (BD) among participants significantly declined from 35.5% to 24.6%, particularly during the initial wave of data collection, with a corresponding decrease in binge drinking days; however, the overall frequency of alcohol consumption remained statistically unchanged. This reduction in binge drinking prevalence is primarily attributed to social distancing measures. Notably, none of the stressors related to COVID-19 were found to correlate with either the prevalence or the severity of binge drinking episodes. In contrast, increased alcohol consumption was associated with distance learning, pre-existing binge drinking behaviors prior to the pandemic, and the use of substances as coping mechanisms. Furthermore, binge drinking was not associated with resilient coping strategies.	8/11
Gohari et al., 2023 Canada [28]	Three cohorts: T1 (2018/19), T2 (2019/20), and T3 (2020/21). In T2, data collection was conducted pre-pandemic (from September 2019 to February 2020, T2a) and post-pandemic (from May to June 2020, T2b),	To investigate changes in alcohol consumption patterns among youth subpopulations during the pandemic. It focuses on analyzing drinking behavior profiles within this demographic and assessing the pandemic’s impact by comparing transitions between different subgroups before and after the pandemic.	Longitudinal cohort study	Using data from an ongoing prospective cohort study (*n* = 5347) in Canadian college students: T2a (*n* = 3447; 57.3% women; aged 12–18); T2b (*n* = 1900; 65.2% women; aged 12–18).	This study examines the prevalence of binge drinking through participants’ self-reported instances of consuming five or more alcoholic beverages in a single occasion over the past year. Participants were classified into four categories: non-binge drinkers, occasional binge drinkers, monthly binge drinkers, and weekly binge drinkers. To identify distinct patterns of alcohol consumption and analyze temporal transitions between these patterns, latent transition analysis (LTA) was employed, utilizing both overall drinking frequency and binge drinking frequency as primary variables.	Individuals were classified as occasional, monthly, or weekly binge drinkers. Before the COVID-19 pandemic, the probability of binge drinking among occasional drinkers was 61%, which decreased to 43% during the early pandemic period. A minor fraction of occasional binge drinkers shifted to more frequent patterns (monthly or weekly), with female occasional drinkers showing a higher likelihood of this transition compared to males. Cohort T2b reported slightly higher binge drinking levels than Cohort T2a, with T2b’s occasional drinkers increasing from 7% to 10%, while in T2a, it rose from 8% to 16%. For Cohort T2a, the probability of maintaining occasional binge drinking was 37%, with transitions to monthly and weekly binge drinking at 22% and 2%, respectively. Overall, the pandemic’s impact on transitions between different alcohol consumption patterns was not statistically significant.	10/11
Gohari et al., 2022 Canada [29]	Four cohorts: T1 (2017/18); T2 (2018/19); T3 (2019/20); T4 (2020/21).	To analyze variations in alcohol consumption across two phases of the pandemic.	Longitudinal cohort study	Longitudinal data (*n* = 14,089) students; age range: 13–18 years; 52.9–63.1% women along the study.	(COMPASS) study (2012–2027). The frequency of binge drinking: “In the last 12 months, how often did you have 5 drinks of alcohol or more on one occasion?”. Prevalence of BD was compared on T1 and T2 versus T3 and T4. A D-I-D model was used to compare changes in the frequency BD between the pre-COVID-19 period to the initial- and ongoing-pandemic periods.	The anticipated increase in binge drinking (BD) frequency from the pre-pandemic period (2018/19) to the initial phase of the COVID-19 pandemic (2019/20) was less pronounced than the changes observed between the 2017/18 and 2018/19 periods across different sex and age groups. However, during the second year of the pandemic, both overall alcohol consumption and binge drinking frequency increased. In the T2/T3 period, male students exhibited a greater decline in BD compared to their female counterparts. Additionally, male students and younger adolescents (aged 12–14) showed a disproportionate increase in alcohol consumption in T3.	10/11
Gohari et al., 2023 Canada [30]	T1 (2018/19); T2 (May to June 2020); T3 (2020/21).	To study changing patterns of alcohol consumption over the pandemic and associations with depression and anxiety symptoms among adolescents.	Longitudinal cohort study	Longitudinal data “COMPASS” (*n* = 1901) students; age range: 13–18 years; 65.3% women and 34.7% men.	Alcohol consumption national surveillance. BD: “In the last 12 months, how often did you have 5 drinks of alcohol or more on one occasion?”. CESD-R-10 and GAD-7. Multilevel logistic regression models were utilized to assess the association between symptoms of depression and anxiety with the likelihood of alcohol consumption.	Symptoms of depression and anxiety exhibited a significant increase over the three-year period, with these changes being influenced by variations in binge drinking (BD). Students with increased depression were more likely to initiate BD before and during the pandemic. Students who initiated BD in T2 reported the greatest increase in depression and anxiety. The proportion of females maintaining, initiating or escalating BD was higher than males, while higher proportions of males reported abstaining from BD than females from T1 to T3. Rates of reduced consumption were similar among males and females from T1 to T2 and T2 to T3.	10/11
Hoots et al., 2023 USA [31]	2009–2021	To examine substance use patterns and understand how substance use among high school students changed before and during the COVID-19 pandemic.	Cross-sectional study	Year 2019: *n* = 13,677 respondents; Year 2021: *n* = 17,232 respondents; age range: 14–18 years; 48.1% women; 51.9% men.	Youth Risk Behavior Survey: prevalences among high school students of current (i.e., previous 30 days) BD.	From 2019 to 2021, the prevalence of BD decreased. Current binge drinking was reported by 10.5% of the sample in 2021. Compared with males, females had a higher prevalence of binge drinking (12.2% versus 9.0%). Males also had a 30% relative decrease in binge drinking from 2019 to 2021.	6/8
Kinouani et al., 2024 France [32]	April to May 2020	To compare self-reported changes in alcohol misuse during the first COVID-19 lockdown between French students and non-students and describe factors associated with alcohol misuse in each subgroup.	Cross-sectional study	The Confins cohort: *n* = 900; age range: 18–≥ 25 years; median age: 24.3 years; 74.3% women, 25.7% men.	BD frequency: “If you drink six drinks of alcohol on one occasion and in a short time, has it happened more frequently since lockdown?”. AUDIT-C, GAD-7, and PHQ-9. Multiple logistic regression was performed to estimate the association between self-reported BD and the variables.	Nine hundred people had BD in the last 7 days, and students reported more suicidal thoughts, high PHQ-9, and GAD-7 scores than non-students. Decrease or no change in BD was more common than an increase. The risk factors explaining an increase in binge drinking frequency were being a tobacco smoker before lockdown and not practicing any physical activity during the last 7 days.	7/8
Mallis et al., 2022 USA [33]	October–December 2020	To identify risk factors (demographic and behavioral) associated with SARS-CoV-2 infection among college students.	Cross-sectional study	Sample of *n* = 679 university students; age range: 20–21 (29%); 78.6% women; 21.4% men.	BD: high, low, or never (five or more alcoholic beverages in a two-hour period (men) and 4 or more in a two-hour period (women). Demographics and academic characteristics, students’ health habits, smoking, alcohol consumption, exercise intensity and duration, and COVID-related questions. Prevalence ratios were calculated.	The majority of individuals reported some binge drinking behavior: 125 participants (18.4%) in the high frequency category and 317 (46.7%) in the low frequency category. SARS-CoV-2 infection was 2.8 times more likely among those who reported a high frequency of BD.	5/8
Miech et al., 2021 USA [34]	February–March 2020 and July–August 2020	To assess if substantial reduction in drug availability will lead to reductions in drug prevalence.	Cross-sectional study	Sample of *n* = 582 12th grade college students; age range: +18 years; 50% women; 50% men.	BD in the past two weeks: “Think back over the LAST TWO WEEKS. How many times have you had five or more drinks in a row? (A “drink” is a bottle of beer, a glass of wine, a wine cooler, a shot glass of liquor, a mixed drink, etc.)”. Multivariable regressions were performed.	Perceived availability of alcohol declined across the two survey waves. Despite these declines, prevalence BD levels did not significantly change. Changes in binge drinking prevalence across the two survey waves differed across the social distancing groups.	5/8
Monzon et al., 2024 Guatemala [35]	May to September 2019 (Wave 1); June to November 2020 (Wave 2).	To assess whether the COVID-19 school shutdown influenced adolescent alcohol (including binge drinking) use.	Cross-sectional study	Wave 1 (*n* = 20,969); Wave 2 (*n* = 1606). Sample of high school students in Guatemala City. Age range: 13–>15; 51.8% women; 48.2% men.	BD: “Have you ever had about 4 or more drinks on one occasion over the prior 30 days?”. Logistic Generalized Estimating Equations to estimate the influence of the COVID-19 lockdown on BD.	Prevalence declined for binge drinking (24% to 13%; *p* < 0.001). Friends’ and household members’ substance use was significantly associated with teenagers’ substance use, and they all significantly decreased in Wave 2.	5/8
Niedzwiedz et al., 2020 United Kingdom [17]	Data from 2015 to 2019, compared to data of the COVID-19 pandemic onset (April 2020)	To investigate the impact of the UK’s COVID-19 lockdown on mental health and health behaviours, as well as whether any observed impacts differed by age, gender, ethnicity, and education level.	Longitudinal study, based on various cross-sectional analysis by waves.	Longitudinal analysis composed by (*n* = 9748) adults, of whom *n* = 655 were aged 18–24 years; 52.0% women; 48% men.	GHQ-12 and AUDIT-C. BD: 6+ drinks in a single sitting on a weekly basis. Prevalence estimates (with 95% CIs) for each outcome were calculated. Multi-level Poisson regression was used.	Psychological distress increased into lockdown with the prevalence rising from 19.4% to 30.6% in April 2020. BD increased from 10.8% in 2017–2019 to 16.2% during the lockdown. The proportion of people drinking four or more times per week increased, as did binge drinking (RR = 1.5; 95% CI: 1.3 to 1.7). Relative risk for BD was significant for women aged 18–24 in comparison to pre-pandemic data.	9/11
Patin et al., 2022 France [36]	May 2020 and May 2021	To assess the progression of healthy behaviors from the pre-COVID-19 period to May 2021.	Retrospective online cross-sectional study.	Sample of (*n* = 6991) university students; mean age: 20.8 (SD = 2.5); 73.4% of women; 26.6% men.	BD in the past week was defined as the consumption of six or more glasses of alcohol on a single occasion. The frequency of BD was classified into three categories: never, occasional, and regular. CES-D8, socio-demographic, and COVID-19-related questions. A multivariate logistic regression model was used.	BD (both occasional and regular) declined in 2020 and 2021 compared to the pre-COVID-19 period. However, a notable increase was observed in May 2021, approaching pre-pandemic levels. This resurgence may be attributed to the relaxation of restrictions, including curfews and partial lockdowns, which allowed for small social gatherings.	6/8
Patrick et al., 2022 USA [37]	April to November 2020	To examine drinking trends (prevalence, frequency, contexts, and reasons) prior to and during the COVID-19 pandemic in 2020 and whether they differed by age and college status.	Longitudinal cohort study	Data from the MTF study (*n* = 29,940) college students. Aged: 18–30 years, with 57.0% from 18 to 24 years; 53.1% women; 46.9% men.	BD: “Think back over the last two weeks. How many times have you had five or more drinks in a row?” Alcohol prevalence and frequency data were obtained through MTF survey. Sensitivity analyses were conducted and full multivariable models including covariates were run.	Prevalence of young adult BD was generally stable from 2015 to 2019 (29.5–31.1%), but 2020 was associated with downward deviation (26.4%) in BD prevalence (young adults). In addition, there was an upward non-significant deviation in BD among drinkers aged 19–30. Among the factors, it was prevalent to drink alone and at home/apartment/dorm, to relax/relieve tension and because of boredom.	8/11
Pelham et al., 2022 USA [38]	June 2020, December 2020, and June 2021	To examine the impact of the COVID-19 pandemic on drinking and nicotine use through June of 2021 in a community-based sample of young adults	Longitudinal cohort study	Sample of *n* = 348 individuals; age range: 18–22 years; mean age: 20 years; 49% women; 51% men.	BD: the number of days in the past 30 days during which individuals consumed ≥ 5 alcoholic drinks (≥4 for females) on an occasion.	There were no statistically significant differences between the pre-pandemic and during-pandemic time points for BD. However, a non-significant reduction was observed in BD in June and December 2020. The pandemic impact on participants’ financial economy did not moderate drinking outcomes.	7/11
Rogés et al., 2021 Spain [39]	October 2019 to February 2020 and June to July 2020.	To identify the changes in binge drinking, the hazardous drinking, the hazardous consumption of cannabis, and the daily smoking of tobacco, in a cohort of 14- to 18-year-old adolescents, due to the COVID-19 pandemic confinement.	Longitudinal cohort study	DESK-COVID-Cohort Wave 2 (*n* = 303); age range: 14–18; 29.7% men and 70.3% women.	BD: “How often do you have ≥6 alcoholic drinks on a single occasion?”. AUDIT-C test. CAST. DESK-COVID-Cohort survey. Cumulative Incidence of Change (IC) and the relative risks (RR) were obtained.	There were 36.3% of participants who consumed alcohol in a binge drinking pattern. Older adolescents attending advanced vocational courses had a significantly (*p* < 0.05) higher risk of binge drinking (RR = 3.21; 95% CI: 1.00–10.34). The overall prevalence of BD decreased from the pre-COVID period (36.3%) to after confinement (5.9%) (*p* < 0.05). The likelihood of BD (RR = 1.46; 95% CI:0.49–4.33) was higher among students with medium and high economic incomes.	9/11
Romano et al., 2022 Canada [40]	May–July 2020	To examine how substance use is associated with perceptions of and adherence to early COVID-19-related public health measures.	Cross-sectional study	Data of a sample (*n* = 7876) in a prospective cohort of Canadian adolescents; age range: 12–19 years; mean age: 15 (SD = 1.6 years); 60% women; 36% men.	COMPASS Student Questionnaire. Participation in BD in the past 12 months; current BD was defined as 5 or more drinks on one occasion at least once per month. Two models were used to estimate how substance use was associated with perceptions and adherence to early COVID-19 restrictions.	There were 11.4% (*n* = 895) of students who reported current binge drinking. Binge drinking was associated with perceptions that restrictions were too strict and with nonadherence, reporting that they did not take COVID-19 restrictions seriously compared to those who did not drink.	7/8
Rubio et al., 2023 Netherlands [41]	April–July 2020	To examine the impact of the initial lockdown on alcohol consumption among university students who engaged in regular binge drinking prior to its implementation.	Cross-sectional study	Sample of (*n* = 7355) university students; mean age: 21.4 (SD = 2.3 years); 60.8% women, 39.2% men.	Participants were categorized as either frequent binge drinkers or regular drinkers according to the response to the specified question: “How often did you drink 6 servings or more on a single occasion before the COVID-19 outbreak?” CES-D8, BRS.	Out of 2065 surveyed students, 35.7% were identified as binge drinkers. During the lockdown period, a majority of regular binge drinkers (70.1%) demonstrated a notable decrease in BD frequency, with a reduction from 12.8% to 7.6%. Factors contributing to increased or sustained alcohol consumption among binge drinkers included older age, a lower baseline alcohol intake prior to the COVID-19 pandemic, greater social interactions, and living independently rather than with parents. Additionally, among regular binge drinkers, males exhibited a significantly greater increase in alcohol consumption compared to females. Furthermore, individuals with pronounced depressive symptoms and lower resilience levels were more likely to increase their alcohol use.	8/8
Serkut Bulut et al., 2021 Turkey [42]	May–June 2020	To explore the relationship between students’ social support systems, health-risk behaviors, and mental/academic well-being of higher-education students in İstanbul during the COVID-19 pandemic.	Cross-sectional study	Sample of (*n* = 2583) higher-education students; mean age = 22.84 (SD = 4.79 years); 65.5% women; 34.5% men.	COVID-19 International Student Well-Being Study. CES-D-8, UCLA-3 Loneliness Scale, Academic Stress Scale, and Academic Satisfaction Scale. Bivariate associations and a binary logistic regression test were conducted.	BD frequency significantly decreased during COVID-19. The frequency of BD was associated with depressive symptoms, loneliness, increased levels of academic stress, and lower academic satisfaction. An accessible and supportive social network was found to be a protective factor against depression. Women and men were almost never binge drinkers before or during COVID-19. During the pandemic, BD was significantly higher among male students compared to females.	6/8
Shapiro et al., 2022 Israel [43]	April–September 2020	To examine the prevalence of risky behaviors among adolescents in Israel during the COVID-19 pandemic and assess the potential impact of the pandemic on the occurrence of these behaviors.	Cross-sectional study	Sample of (*n* = 1020) adolescents; age range: 15–18 years; mean age = 16.73 (SD = 0.99 years); 57.3% women; 42.7% men.	BD: “In the past 30 days, how many times have you drank five drinks of alcohol or more within a period of a few hours?”. HBSC survey, FAS, and MSPSS. Binary logistic regression models were used.	In the studied population, BD emerged as the most common risky behavior, reported by 33.8% of participants. A significant correlation was observed between BD and broader patterns of risky behavior, including tobacco use and cannabis consumption. During the pandemic, the majority of respondents (55.2%) exhibited no change in the frequency of their drinking behavior. Conversely, 14.4% reported initiating or increasing their BD frequency, while 15.9% noted a decrease in alcohol consumption. Factors, such as low family support, high socioeconomic status, advanced age, male gender, and elevated emotional distress, were identified as predictors of BD. Notably, support from friends, levels of physical activity, and COVID-19-related restrictions did not significantly influence binge drinking behavior.	7/8
Sharma et al., 2022 USA [44]	April 2020–March 2021	To study the associations between demographic factors, psychological distress, and changes in alcohol use before and after the onset of COVID-19 in adolescents and young adults.	Longitudinal cohort study	Sample of (*n* = 2216) individuals; age range: 16–20 years, with 40.7% from 16 to 18 years; 81% women and 19% men.	Risk of increased alcohol consumption: binge drinking (never, less than monthly, monthly, weekly, or daily), number of drinks, and drinking regularity. PHQ-9 and GAD-7. Logistic regression models were used.	No changes in BD were reported by 74.6% of the sample, and 17.0% reported an increase in BD. Older age, college students, increased anxiety, and smoking status showed significant associations during the pandemic.	8/11
Sylvestre et al., 2022 Canada [45]	December 2020–June 2021	To assess changes in substance use among young adults before and during the COVID-19 pandemic, with a focus on identifying factors that contribute to the initiation or increase of substance use during this time.	Longitudinal cohort study	Longitudinal investigation of (*n* = 1294) youth from 1999 to 2021; a sample of (*n* = 704) was obtained during the pandemic. Mean age: 33.6 (SD = 0.6 years); 58.2% women; 41.8% men.	BD: consuming ≥ 5 alcoholic beverages on one occasion. Data on the utilization of cannabis, alcohol, combustible cigarettes, e-cigarettes, and binge drinking prior to the pandemic were gathered. Modified Poisson regression was used.	Amid the pandemic, 7.9% of respondents reported weekly occurrences of binge drinking, and 12% reported daily occurrences. The rate of individuals who either discontinued or diminished their binge drinking behavior was significantly higher during this period, at 53.5%, particularly among those aged between 24.0 and 30.6 years. Additionally, low socioeconomic status, mental health conditions, and solitary living arrangements were associated with an elevated likelihood of engaging in weekly or daily binge drinking.	7/11
Tavolacci et al., 2021 France [46]	March–May 2020	To assess the modifications in health-related behaviors among students enrolled at a university in France throughout the COVID-19 lockdown	Cross-sectional study	Sample of (*n* = 3671) university students; mean age: 20.9 (SD = 2.47 years); 72.9% women; 27.1% men.	BD is operationally defined as the consumption of six or more alcoholic beverages in one sitting, with frequency classified into four categories: never, occasional, weekly, and daily. CESD-8, academic, and COVID-related data. A multivariable logistic regression model was used.	The data revealed a significant decrease in binge drinking prevalence from 35.9% before the COVID-19 pandemic to 9.3% during the pandemic. Only 3.1% of participants reported an increase, predominantly among male students who had never lived with their parents and had higher CESD-8 scores. Factors linked to the reduction in binge drinking included enrolment in healthcare programs, being in the second year or beyond of their studies, returning to live with parents, living alone during the pandemic, awareness of local COVID-19 cases, and concerns about severe health risks from the virus.	7/8
Tholen et al., 2022 Belgium [47]	April–May 2020	To examine associations between pandemic-related stressors, psychosocial distress, and self-reported alcohol, tobacco, and cannabis use before and during the first wave of the pandemic.	Cross-sectional study	Sample of (*n* = 18,346) higher education students; age range: 17 to 24; 75% women and 25% men.	BD is defined as the consumption of six or more alcoholic beverages during a single event. To analyze the data, multinomial logistic regression techniques were employed.	A total of 2289 (12.5%) students declared the use of BD during COVID-19. A total of N = 7335 students declared themselves as no binge drinkers. BD decreased during the pandemic (87.4%) due to limited social gatherings and low economic status. Returning to the parental home is linked to reduced BD, whereas depressive symptoms and psychosocial distress are associated with increased BD. Perceived threat and academic stress were also associated with increased BD.	7/8
Vallentin-Holbech et al., 2023 Denmark [48]	August 2020 and November 2020	To investigate the changes in hazardous alcohol consumption, social interactions, and overall well-being among first-year Danish students during the second wave of the COVID-19 pandemic.	Cross-sectional study	Sample of (*n* = 352) Danish students in secondary school; age range: 15–20 years; mean age: 16.8 (SD = 0.74 years); 66.8% women and 33.2% men.	BD: the number of days. Alcohol use was measured using the Timeline Follow-back; alcohol-related negative consequences were assessed using the RAPI23. COVID-19 variables were measured using the PHQ-4. Multilevel regression models were used.	During COVID-19-pandemic, students decreased the frequency and quantity of binge drinking reduced from a mean of 2.35 ± 2.35 days a month to 1.46 ± 2.59 days. Decrease in BD was associated with attending fewer parties. Variations in mental health and experiences of loneliness did not correlate with a reduction in hazardous alcohol use.	6/8
van Hooijdonk et al., 2022 Netherland [49]	April–June 2020	To examine the influence of COVID-19 on trends in weekly smoking, binge drinking, and cannabis use among Dutch university students, as well as associated characteristics related to the pandemic.	Cross-sectional study	Sample of (*n* = 9967) university students; mean age: 22.0 (SD = 2.6 years); 70.3% women and 29.7% men.	BD before and during COVID-19: drinking ≥ 6 glasses on a single occasion. Multivariate logistic regression analyses were used.	The prevalence of weekly binge drinking was higher among males than females both before and during the initial COVID-19 lockdown. A study of 6884 students indicated a significant decline in weekly binge drinking from 27.8% to 13.9% during the pandemic. Among those who did not binge drink prior to COVID-19, 6.2% reported an increase in such behavior. Key factors associated with increased risk included being male, not living with parents, being an undergraduate student, having limited financial resources, and lower adherence to COVID-19 safety measures. Older age was linked to a reduced likelihood of binge drinking before the pandemic but a higher likelihood during it. Complicated relationship status also raised the probability of binge drinking during COVID-19, whereas strict adherence to safety measures correlated with lower binge drinking rates.	6/8
Vasconcelos et al., 2021 Portugal [50]	April–May 2020; October–November 2020.	To examine the impact of the COVID-19 pandemic on college students’ alcohol consumption habits by evaluating how personal characteristics, emotional states, lifestyle choices, and social context influenced their alcohol use and binge drinking behaviors.	Longitudinal cohort study	Sample of 146 college students; mean age: 19.5 (SD = 1.5 years); age range: 17–26 years; 81% women and 19% men.	Regular binge drinkers, defined as individuals who consume five or more alcoholic beverages on a single occasion at least once a month, as well as infrequent binge drinkers and non-binge drinkers. AUDIT, PACS, and DASS-21. Linear regressions were implemented. Linear mixed-effects models were estimated.	The alcohol consumption of regular binge drinkers (BDs) significantly decreased from the pre-lockdown period (M = 10.9) to the lockdown period (M = 4.8; *p* < 0.001) and further declined post-lockdown (M = 2.2; *p* < 0.001). Regular BDs maintained lower alcohol intake than usual even after the cessation of isolation restrictions. During the lockdown, significant differences in weekly binge drinking were observed only between the non-binge drinkers and regular binge drinkers. No significant interactions were found between stress, anxiety, depression, and drinking group that could explain variations in alcohol consumption. Factors positively correlated with increased alcohol intake during the lockdown included a history of drunkenness, heightened cravings, and cohabiting with friends. The COVID-19 mitigation measures and social distancing likely disrupted the typical social contexts that facilitate binge drinking.	10/11
Zysset et al., 2022 Switzerland [51]	April 2020–June 2021	To examine increase in alcohol consumption, single and multiple binge drinking, and associated factors in students during the lockdown and post-lockdown periods.	Longitudinal cohort study	Sample of *n* = 947 university students; mean age: 27.0 (SD = 6.5 years); 75.8% women and 24.2% men.	BD: any binge drinking in the past 30 days (≥ 5 drinks on one or more occasion); “Think back again over the last 30 days. How many times (if any) have you had five or more drinks on one occasion?”. GAD-7, ASKU, BRCS, and Oslo-3 Social Support Scale. Generalized Estimating Equations models were used.	There were 26% of the students who engaged in BD during the pandemic. Higher anxiety scores were associated to binge drinking. Additional factors associated with BD were male gender, younger age, higher perceived social support, and not living with parents. University students who consumed more alcoholic drinks at baseline were more likely to report at least one binge drinking event in the past 30 days. BD was not associated with lower resilience or self-efficacy.	9/11

Table legend: ASKU: General Self-Efficacy Expectations Short Scale; AUDIT: Alcohol Use Disorder Identification Test; BD: binge drinking; BRCS: Brief Resilient Coping Scale; BRS: Brief Resilience Scale; CAST: Cannabis Abuse Screening Test; CES-D8: Centre for Epidemiologic Studies Depression Scale; CESD-R-10: 10-item Center for Epidemiologic Studies Depression Scale Revised Scale; DASS-21: Depression Anxiety Stress Scale-21; D-I-D: difference-in-difference; FAS: Family Affluence Scale; GAD-7: Generalized Anxiety Disorder 7-item Scale; HBSC: Health Behavior in School-Aged Children; HRB: Health Risk Behaviors; IC: Cumulative Incidence of Change; MSPSS: Multidimensional Scale of Perceived Social Support; MTF: Monitoring the Future; PACS: Penn Alcohol Craving Scale; PHQ-4: Patient Health Questionnaire for Depression and Anxiety 4 items; PHQ-9: Patient Health Questionnaire 9 items; RAPI23: Rutgers Alcohol Problem Index 23 items; RR: relative risk; SDBQ-L: Short Diet Behavior Questionnaire for Lockdowns; UCLA-3: University of California, Los Angeles-3 item Loneliness Scale.

## Data Availability

The original contributions presented in this study are included in the article/Supplementary Materials. Further inquiries can be directed to the corresponding authors.

## Supplementary Materials

The following supporting information can be downloaded at: https://www.mdpi.com/article/10.3390/jcm14051546/s1, Table S1. PRISMA checklist; Table S2. JBI score for cross-sectional studies; Table S3. JBI score for cohort studies.

## Abbreviations

The following abbreviations are used in this manuscript:

BD	binge drinking
COVID-19	Coronavirus Disease 2019
JBI	Joanna Briggs Institute
PRISMA	Preferred Reporting Items for Systematic Reviews and Meta-Analyses
WHO	World Health Organization
SARS-CoV-2	Severe Acute Respiratory Syndrome Coronavirus 2
